# A History of Dental and Oral Science in America

**Published:** 1876-12

**Authors:** 


*A History of Dental and Oral Science in America.*—Publisher Samuel S. White, Philadelphia, 1876.

This work, which comprises some two hundred and seventy pages, was prepared under the direction of the American Academy of Dental Science, of Boston, for the Centennial Exhibition at Philadelphia, and professes to be a history of the dental profession in this country for the past one hundred years, and a standard of reference comprehensive and complete in detail. Application was made to the members of the dental profession to furnish the necessary information for such a work, but the greater portion was received, we learn, in response to personal requests by James E. Dexter, of New York, who was engaged to carry out the task. Expression of opinion was scrupulously avoided and no unnecessary criticism of persons, books, or methods indulged in. After an introductory relating to the art as practiced by the ancients, the subjects of Mechanical Dentistry, Operative Dentistry, Diseases, Injuries, and Natural Defects, of the Mouth, are treated, followed by Dental Associations, Dental Colleges, Dental Legislation, Dental Literature, and Dental Education.

Although the time at the disposal of the committee and compiler was limited, necessitating its hasty preparation, yet this work is an interesting and useful one, and well worth a place in the libraries of the dental practitioners throughout the country.

